# Prepregnancy Obesity Versus Excessive Gestational Weight Gain: A Comparative Evaluation of Adverse Outcomes

**DOI:** 10.3390/jcm15176858

**Published:** 2026-09-04

**Authors:** Samuel Schultz, David Krantz, Matthew J. Blitz, Ashley N. Battarbee, Moti Gulersen

**Affiliations:** 1 Division of Maternal-Fetal Medicine, Department of Obstetrics and Gynecology, Sidney Kimmel Medical College, Thomas Jefferson University, Philadelphia, PA 19107, USA; samuel.schultz@jefferson.edu; 2 Northwell Health Laboratories, Northwell Health, New Hyde Park, NY 11042, USA; dkrantz@northwell.edu; 3 Division of Maternal-Fetal Medicine, Department of Obstetrics and Gynecology, South Shore University Hospital, Bay Shore, NY 11706, USA; 4 Department of Obstetrics and Gynecology, Center for Women’s Reproductive Health, University of Alabama at Birmingham, Birmingham, AL 35294, USA; anbattarbee@uabmc.edu

**Keywords:** obese, body mass index, gestational weight gain, maternal morbidity, neonatal morbidity

## Abstract

**Background/Objectives:** Obesity and excessive gestational weight gain (EGWG) are known risk factors for adverse outcomes in pregnancy, but the comparative risk is less well defined and increasingly relevant in an era of increasing use of weight loss medications. We aimed to determine whether EGWG was associated with adverse maternal and neonatal outcomes, compared with prepregnancy obesity. **Methods:** Multicenter retrospective cohort study of pregnant patients who delivered a singleton within a single university health system from 2019–2024. Patients with missing prepregnancy body mass index (BMI) data, BMI < 18.5 kg/m^2^, or pregestational diabetes were excluded. Logistic regression with propensity score weights and g-computation were used to evaluate if the incidence of several adverse maternal and neonatal outcomes were impacted by prepregnancy obesity and gestational weight gain (GWG). *p* values were adjusted for multiple comparisons. **Results:** Among the 129,615 pregnancies included, 28,516 (22.0%) had prepregnancy obesity, 36,943 (28.5%) were overweight prior to pregnancy, and 64,156 (49.5%) had a normal BMI prior to pregnancy. When compared to patients with obesity, patients with normal prepregnancy BMI and EGWG had a lower risk of adverse maternal and neonatal outcomes, including gestational diabetes, gestational hypertension, preeclampsia, eclampsia, cesarean delivery, and postpartum hemorrhage, as well as large and small for gestational age birthweight, stillbirth, neonatal intensive care unit admission, neonatal mechanical ventilation, respiratory distress syndrome, intraventricular hemorrhage, necrotizing enterocolitis, and sepsis. Beneficial effects were attenuated for patients with overweight prepregnancy BMI or for larger EGWG. **Conclusions:** Lower prepregnancy BMI, even with the potential unintended consequence of EGWG, may be more favorable than untreated prepregnancy obesity with regard to perinatal outcomes.

## 1. Introduction

Obesity, defined as a body mass index (BMI) of 30 or greater, is common, occurring in 39.7% percent of reproductive age individuals in the United States (U.S.) [1]. As a medical condition, it has been associated with increased rates of several adverse pregnancy outcomes including miscarriage and stillbirth, congenital anomalies, hypertensive disorders of pregnancy, gestational and pregestational diabetes, fetal growth restriction (FGR), and large for gestational age (LGA) neonates [1,2,3]. While there are many proposed theories behind the pathophysiology of this association, fundamental metabolic and inflammatory changes associated with obesity are thought to be the main drivers of these outcomes in pregnancy [2,3]. Another weight-based concern during pregnancy is excessive gestational weight gain (EGWG), defined as total pregnancy weight gain that exceeds the upper limit of the National Academy of Medicine (NAM) (previously known as the Institute of Medicine) recommended range for a patient’s prepregnancy BMI category [4]. EGWG has been linked to increased neonatal birth weight and maternal postpartum weight retention, as well as increased rates of similar pregnancy complications and adverse neonatal outcomes associated with obesity during pregnancy [5,6,7].

Prepregnancy management of obesity, with surgical or nonsurgical methods, can help decrease the incidence of obesity in pregnancy and reduce the risk of associated comorbidities [1]. Glucagon-like peptide-1 receptor agonists (GLP-1 RAs) have emerged as a popular treatment strategy for patients with obesity, as their use can result in a weight loss range of 5.8–22.1% [8]. However, GLP-1 RAs are currently contraindicated in pregnancy, and thus an important consideration to prepregnancy GLP-1 RA use is rebound weight gain after discontinuation, which also increases the risks of adverse outcomes in pregnancy [9,10]. The impact of GLP-1 RA use prior to pregnancy is currently being investigated with mixed results as to whether they improve or worsen obstetric and neonatal outcomes [11,12,13].

Whether entering pregnancy with a lower prepregnancy BMI followed by EGWG is associated with better maternal and neonatal outcomes than entering pregnancy with obesity is not well understood. Therefore, we sought to estimate the adjusted risks of adverse maternal and neonatal outcomes associated with these two clinical scenarios.

## 2. Materials and Methods

This was a multicenter retrospective cohort study of pregnant patients who delivered a singleton at ≥20 weeks’ gestation in 7 hospitals within a single university health system (Northwell Health, NY, USA) between 1 January 2019 and 31 December 2024. Patients with missing prepregnancy BMI data, BMI < 18.5 kg/m^2^, or pregestational diabetes were excluded.

The primary objective was to estimate how the risk of adverse maternal and neonatal outcomes would change under hypothetical scenarios in which patients who entered pregnancy with obesity instead entered pregnancy with a lower prepregnancy BMI, with or without EGWG. Because these scenarios cannot be evaluated in a randomized study, we used a counterfactual causal inference framework consisting of three sequential steps. First, propensity score weighting was used to balance baseline characteristics between comparison groups. Second, weighted logistic regression models were fit to estimate the relationship between prepregnancy BMI, GWG, and each outcome. Third, g-computation was used to estimate the adjusted outcome risks under alternative prepregnancy BMI and GWG scenarios while holding all other patient characteristics constant.

We evaluated multiple adverse maternal and neonatal outcomes. Adverse maternal outcomes included gestational diabetes (GDM), gestational hypertension, preeclampsia, eclampsia, shoulder dystocia, operative vaginal delivery, cesarean delivery, and postpartum hemorrhage. Adverse neonatal outcomes included stillbirth, LGA birthweight, small for gestational age (SGA) birthweight, neonatal intensive care unit (NICU) admission, use of mechanical ventilation, respiratory distress syndrome (RDS), intraventricular hemorrhage (IVH), necrotizing enterocolitis (NEC), hypoxic ischemic encephalopathy (HIE), neonatal sepsis, and umbilical artery pH < 7. Adverse outcomes were defined according to institutional guidelines and identified by utilizing the International Classification of Diseases, 10th Revision, Clinical Modification (ICD-10) codes, and clinical documentation derived from the inpatient medical record (EMR) system (Sunrise Clinical Manager, Veradigm, Chicago, IL, USA). Baseline demographic data were also extracted from the EMR including maternal age, self-reported race and ethnicity, insurance type, parity, and chronic hypertension.

Prepregnancy and delivery BMI data were self-reported at the time of delivery hospitalization and classified as normal (18.5–24.9 kg/m^2^), overweight (25.0–29.9 kg/m^2^), and obesity (≥30.0 kg/m^2^). EGWG was defined as exceeding the recommended upper limit of total weight gain for a given prepregnancy BMI category using the NAM GWG guidelines: >35 lbs (15.9 kg) for patients who had a normal BMI, >25 lbs (11.3 kg) for patients who were overweight, and >20 lbs (9.1 kg) for patients with obesity [4].

Categorical variables were compared using Rao-Scott χ^2^ tests and continuous variables using design-adjusted analysis of variance to account for multiple pregnancies within the same patient.

Propensity score weights were estimated using multinomial logistic regression with the average treatment effect among the treated (ATT) approach so that pregnancies with prepregnancy obesity remained the reference population. Covariates included maternal age, race and ethnicity, insurance status, parity, and chronic hypertension. Separate weighting schemes were developed for each planned comparison (described below). Covariate balance after weighting was assessed using standardized mean differences and effective sample size.

Weighted logistic regression models with robust standard errors were then fit for each maternal and neonatal outcome, accounting for the propensity score weights and clustering of multiple pregnancies within the same patient. Models included prepregnancy BMI category, GWG category, and their interaction. Firth penalized logistic regression was used when outcome frequencies were too small for stable maximum likelihood estimation.

G-computation was subsequently used to estimate adjusted relative risks by calculating predicted outcome risks under the observed exposure and then recalculating those risks after substituting alternative prepregnancy BMI and GWG categories while holding all other patient characteristics unchanged. Relative risks, 95% confidence intervals, and *p* values were derived by comparing these predicted risks, with standard errors estimated using the delta method.

Four prespecified counterfactual comparisons were performed. First, we estimated the association of lowering prepregnancy BMI from obesity to overweight or normal, while holding GWG constant. Second (primary analysis), we estimated the association of lowering prepregnancy BMI while simultaneously assuming EGWG, compared with prepregnancy obesity regardless of GWG. Third, we compared overweight or normal prepregnancy BMI with EGWG to prepregnancy obesity with recommended GWG. Finally, an exploratory analysis evaluated whether the degree of EGWG (1–9 lb [0.5–4.1 kg], 10–19 lb [4.5–8.6 kg], or ≥20 lb [≥9.1 kg] above NAM recommendations) modified these associations.

A sensitivity analysis excluding deliveries before 37 weeks of gestation was performed to minimize confounding related to prematurity and duration of pregnancy available for weight gain. Because 19 outcomes were evaluated, *p* values were adjusted using the Benjamini–Hochberg procedure. Statistical significance was defined as *p* < 0.05.

The institutional review board approved this study as minimal-risk research using data collected for routine clinical practice and waived the requirement for informed consent.

## 3. Results

Among the 150,023 pregnancies during the study period, there were missing data on prepregnancy BMI data and GWG (20,094), public insurance status (173) and parity (259). There were 118 patients with multiple fields missing. A total of 129,615 patients were eligible for analysis, of which 28,516 (22.0%) had a prepregnancy BMI classified as obesity, 36,943 (28.5%) classified as overweight, and 64,156 (49.5%) classified as normal. Among all of the covariates and outcomes there was good balance between those pregnancies with BMI and GWG and those without (Table S1). As a result, complete case analysis was performed. In addition, there were 38 pregnancies without outcome information on gestational hypertension, preeclampsia, eclampsia, LGA, and SGA, as well as 48,995 pregnancies with missing information on umbilical artery pH. These pregnancies were excluded only for the analyses related to the specific outcome being evaluated.

Baseline demographics compared among the three groups are presented in Table 1. Patients with prepregnancy obesity were more commonly parous, non-Hispanic black, used public insurance, and had a higher prevalence of chronic hypertension compared to patients who were overweight or had a normal BMI prior to pregnancy (Table 1). Patients who had a normal BMI prior to pregnancy more commonly had the recommended GWG in pregnancy, while patients with prepregnancy obesity or overweight prior to pregnancy more commonly exceeded the recommended GWG (Table 1).

After propensity score weighting, covariates were balanced between the three prepregnancy BMI groups for each of the GWG categories while effective sample size remained large (Supplementary Tables S2–S4). Covariates were also balanced when weighting was based on all combinations of prepregnancy BMI and GWG using a single group for EGWG (Supplementary Table S5) or three separate categories of EGWG (Supplementary Table S6).

**Figure 1:**
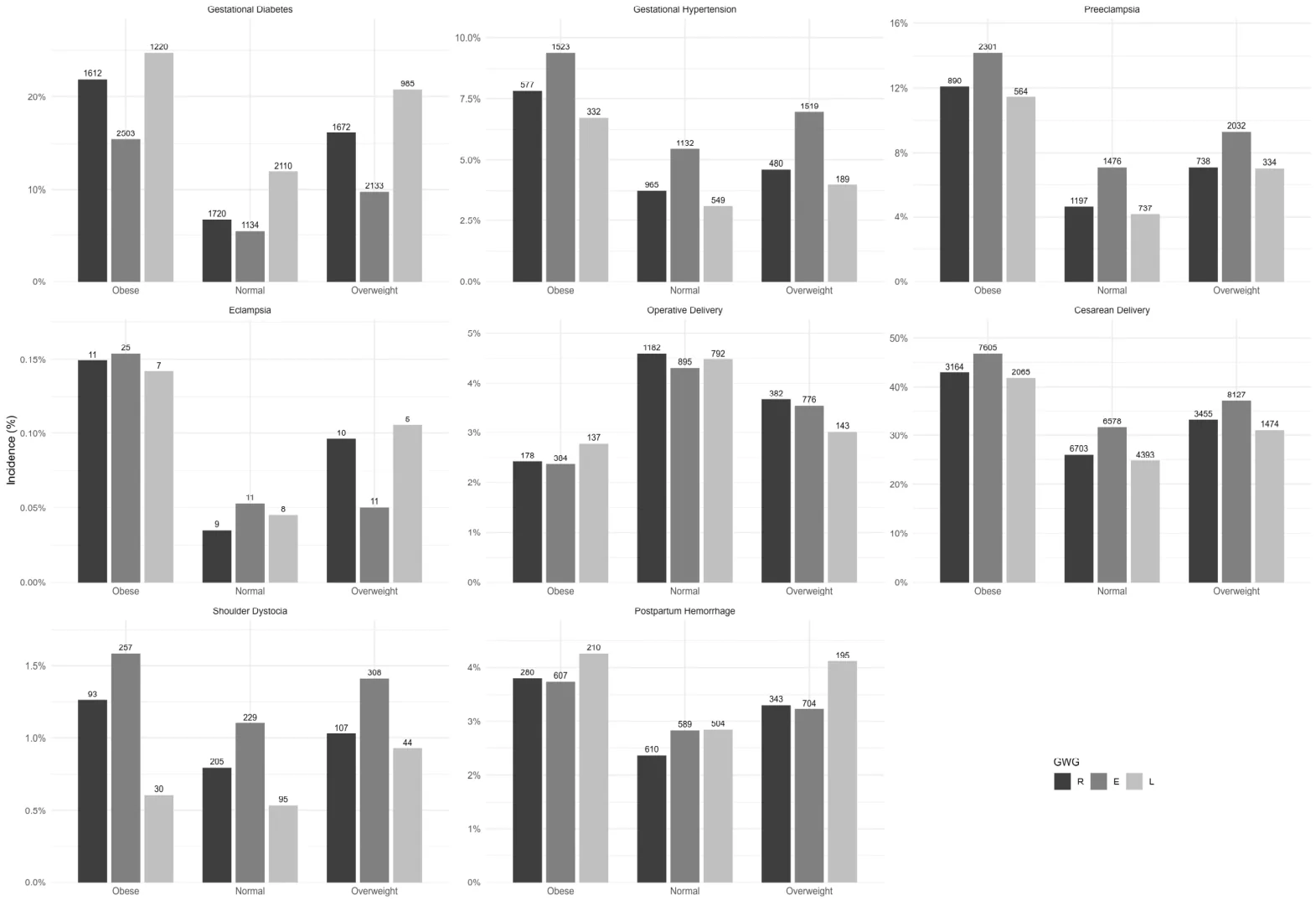
Incidence of adverse maternal outcomes compared between BMI categories and GWG categories.

**Figure 2:**
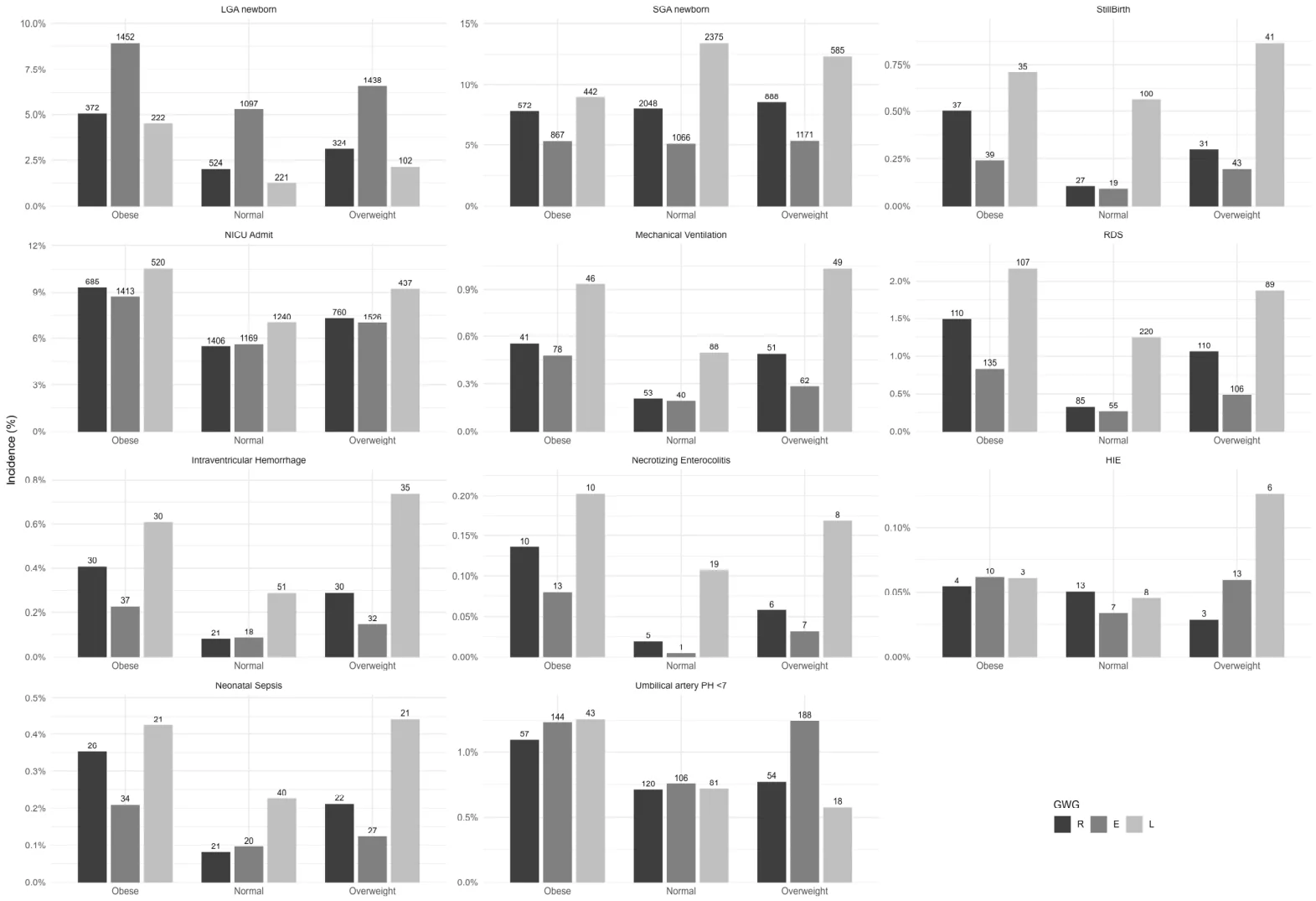
Incidence of n adverse neonatal outcomes compared between BMI categories and GWG categories.

Figure 1 and Figure 2 show the incidence of each maternal and neonatal outcome based on BMI and GWG category. When GWG held steady, compared to prepregnancy obesity, a normal prepregnancy BMI was associated with a reduced risk of all maternal outcomes and neonatal outcomes except for SGA (which showed an increased risk) and HIE (Table 2). While for patients who had prepregnancy overweight BMI, the magnitude of the change was less and was no longer significant for the maternal outcome of shoulder dystocia. In addition, the associated risk reduction for neonatal outcomes were no longer significant except for LGA, NICU admission, RDS, and NEC, while the association with SGA remained significantly increased (Table 2).

In the primary analysis, the impact of a normal prepregnancy BMI with EGWG was associated with a reduced risk of maternal adverse outcomes including GDM, gestational hypertension, preeclampsia, eclampsia, cesarean delivery, and postpartum hemorrhage while increasing the risk of operative vaginal delivery (Table 3). When similar comparisons were done for patients entering pregnancy with an overweight BMI with EGWG, the risk of maternal adverse outcomes was also significantly reduced (although not as much as for normal BMI), including GDM, gestational hypertension, preeclampsia, eclampsia, cesarean delivery, and postpartum hemorrhage. The risk of operative vaginal delivery was increased, although not as much as in the normal BMI group (Table 3).

As seen with the maternal outcomes, entering pregnancy with a normal BMI with subsequent EGWG was associated with a reduced risk of adverse outcomes including LGA, SGA, stillbirth, NICU admission, mechanical ventilation, RDS, IVH, NEC, neonatal sepsis, and umbilical artery pH < 7. When similar comparisons were performed for patients entering pregnancy with an overweight BMI with EGWG, the risk of neonatal adverse outcomes was also reduced (although not as much as for normal BMI), including SGA, stillbirth, NICU admission, mechanical ventilation, RDS, IVH, NEC, and neonatal sepsis. However, the risks of LGA and umbilical artery PH < 7 were no longer significantly reduced. Figures S1 and S2 show the average predicted risk for each combination of prepregnancy BMI and GWG when utilized in the logistic regression formulas and applied to subjects with obesity.

Similar associations were observed when comparing prepregnancy normal or overweight BMI with EGWG to prepregnancy obesity and recommended GWG, with the exception of LGA. For LGA, there was no change in risk when lowering BMI to normal with EGWG (Table 3), while there was an increase in risk when lowering BMI to overweight with EGWG.

The relative risk comparing prepregnancy normal BMI with EGWG of 1–9 lbs, 10–19 lbs, and ≥20 lbs to obesity with recommended GWG was significantly different for the following maternal (GDM, gestational hypertension, preeclampsia, eclampsia, and cesarean delivery) and neonatal (LGA and SGA) outcomes (Table 4). Generally, larger EGWG was associated with less risk reduction than more moderate amounts of EGWG, although the reverse was true for SGA and the risk of LGA increased with larger EGWG. For prepregnancy overweight BMI, similar trends were found for maternal and neonatal outcomes with the exception of eclampsia, shoulder dystocia, and NICU admission. Eclampsia did not show a significant trend, shoulder dystocia showed an increase, and NICU admission showed a decrease in the magnitude of the change in risk for increasing EGWG (Table 5).

In our sensitivity analysis excluding preterm births <37 weeks, we compared prepregnancy normal BMI with EGWG to prepregnancy obesity with any GWG. The risk of the adverse maternal outcomes of GDM, gestational hypertension, preeclampsia, eclampsia, cesarean delivery, shoulder dystocia, and postpartum hemorrhage were significantly lower, while operative vaginal delivery was significantly higher (Table 6). The risk of neonatal outcomes such as LGA, SGA, NICU admission, mechanical ventilation, and umbilical artery PH < 7 were significantly lower (Table 6). Comparing prepregnancy overweight BMI with EGWG to prepregnancy obesity with any GWG, the risk of the adverse maternal outcomes of GDM, gestational hypertension, preeclampsia, eclampsia, and cesarean delivery were lower, while the risk for operative vaginal delivery was higher. For neonatal outcomes, SGA and NICU admission risk were lower (Table 6).

## 4. Discussion

Entering pregnancy without obesity, and particularly a normal BMI, was associated with a reduction in the risk of adverse outcomes, and this effect persisted even with EGWG. In addition, when comparing prepregnancy obesity with any GWG with an overweight prepregnancy BMI (rather than normal) and EGWG, there was still a reduction in adverse outcomes, though the effect was attenuated. These findings suggest that treatment of prepregnancy obesity in order to enter into pregnancy without obesity, even with the potential unintended consequence of EGWG, may be more favorable with regard to perinatal outcomes than untreated prepregnancy obesity. This effect can particularly be seen if BMI can be brought into the normal range and extreme EGWG can be avoided.

Our results largely align with others that compare maternal and neonatal outcomes between patients who have prepregnancy obesity and patients with EGWG. Two studies by McLaren et al. review this topic: one investigating maternal outcomes and the other comparing neonatal outcomes [14,15]. Obesity with and without EGWG was associated with increased rates of GDM, hypertensive disorders of pregnancy, cesarean sections for maternal outcomes, and increased rates of preterm delivery, LGA infants, low APGAR scores, NICU admissions, and immediate need for ventilatory support [14,15]. These studies, however, used prepregnancy normal BMI and recommended GWG as their reference group, while our study used patients with prepregnancy obesity as the reference group. Similarly, in our study, prepregnancy obesity was associated with worse neonatal and obstetric outcomes. However, our study contributes to the literature by adding more details regarding neonatal outcomes, specifically showing statistically significant associations with worse outcomes for patients with obesity in terms of stillbirth, neonatal sepsis, and RDS. A more detailed evaluation of neonatal risks adds emphasis to the existing understanding regarding obesity and maternal outcomes. Additionally, it may provide a more realistic estimation of the tradeoff of rebound weight gain after discontinuation of weight loss medications such as GLP-1 RAs for prepregnancy treatment of obesity.

There are limited data specifically evaluating the impact of prepregnancy GLP-1 RA use and subsequent discontinuation during pregnancy [16]. A retrospective cohort study by Maya et al. found that patients who discontinued their GLP-1RAs before or in early pregnancy had a higher risk of EGWG compared to unexposed patients [11]. Their study also concluded that the discontinuation group also had a higher risk of preterm delivery, GDM, and hypertensive disorders of pregnancy [11]. While their study showed adverse maternal and neonatal outcomes with rebound weight gain after exposure to GLP-1 RAs, both the exposed and unexposed cohorts were made up primarily of individuals with obesity [11]. Our retrospective review suggests that if prepregnancy treatment of obesity is successful at achieving a normal or overweight BMI, the benefits may outweigh the risks of rebound weight gain during pregnancy.

Other recent studies have investigated the concept of lower GWG for patients with obesity in pregnancy or even weight loss. A cohort study by Johansson et al. investigated the value of GWG below the NAM guidelines in pregnancy [17]. The results showed that lower GWG in pregnancy for patients with class 1 obesity (BMI of 30 to 34.9 kg/m^2^) to class 2 obesity (BMI of 35.0 to 39.9 kg/m^2^) was not associated with an increased risk of adverse outcomes [17]. For patients with class 3 obesity (BMI > 40 kg/m^2^) GWG values below the NAM guidelines and weight loss were associated with a reduced risk of adverse outcomes [17]. While our study did not investigate the utility of weight loss in pregnancy, it did show that prepregnancy BMI optimization can be attenuated by the extremes of EGWG, adding to the evidence that weight management before and during pregnancy may be necessary to reduce the risk of adverse pregnancy outcomes.

This study adds to the consensus that prepregnancy obesity is associated with adverse maternal and neonatal outcomes, while also positing that starting pregnancy without obesity may be less detrimental than obesity even with EGWG. Some possible mechanisms that may contribute to worse outcomes in the prepregnancy obesity group could be the increased insulin resistance and inflammation associated with the metabolic changes that often accompany obesity [1,2,3]. While some of these effects may occur in patients without obesity and EGWG at some point in pregnancy, for patients with prepregnancy obesity the effect may be more pronounced.

As obesity management outside of pregnancy becomes more robust with the use of GLP-1RAs, in addition to existing medical and surgical management, more options may be available for patients to lower their BMI prior to pregnancy. This, in addition to nutrition optimization beyond weight management, may help clinicians optimize patient health status prior to pregnancy to help ensure improved outcomes. Even with potential rebound weight gain, it still may be more beneficial to start pregnancy with a lower BMI than a higher one. More research is needed, however, in how best to optimize patient’s nutritional and BMI status prior to conception.

While prepregnancy obesity has been shown to be linked to adverse obstetric and neonatal outcomes, there are still several questions that require more investigation. Though the American College of Obstetricians and Gynecologists (ACOG) follows the NAM Weight Gain Recommendations for Pregnancy, this study adds new information about the potential benefits of a normal BMI prior to the start of pregnancy instead of weight optimization during pregnancy. More research is needed for further recommendations on how to manage the metabolic and inflammatory factors associated with obesity that contribute to these adverse outcomes.

Additionally, though our study addresses EGWG in patients without obesity, it cannot address the nutritional status of patients prior to pregnancy (e.g., comparing patients without obesity who recently had obesity but lost weight as part of a weight loss program versus patients without obesity who were not part of a weight loss program). Although ACOG recommends BMI optimization as part of pregnancy planning, optimizing nutritional status is complex and requires more investigation [18,19]. Future research regarding the use of widely available GLP-1 RAs may also reveal new information about other potential benefits beyond weight loss.

This study has several strengths. We utilized a multicenter cohort with a robust sample size of over 125,000 patients. Our patient population is diverse in terms of maternal demographics and social determinants of health, which makes our findings generalizable. A propensity score-weighted analysis was utilized to help match participants and compare outcomes for a more comprehensive comparison between demographic groups. In addition, we used ATT weighting to identify the impact on risk in the population with obesity, which is the group most likely to undergo treatment. This analysis factored in covariates, such as maternal age, parity, race and ethnicity, insurance type, and chronic hypertension, in order to reduce the risk of confounding. Lastly, we performed a sensitivity analysis excluding preterm births <37 weeks of gestation to account for adverse outcomes that may have been attributed to prematurity and to minimize confounding related to time for weight gain during pregnancy.

This study also has several limitations. Despite the use of propensity scoring, there exists the possibility of unmeasured confounding, such as socioeconomic status, healthcare bias, and access to nutritional support prior to and during pregnancy. Additionally, there may be reporting bias in the estimation of prepregnancy BMI. We also do not have detailed data about weight fluctuations prior to pregnancy, which may be important in future studies to understand how metabolic or inflammatory changes prior to pregnancy affect the risk of adverse outcomes.

## 5. Conclusions

Lower prepregnancy BMI with any GWG is associated with a reduction in the risk of adverse maternal and neonatal outcomes, even if the lower prepregnancy BMI is associated with EGWG. However, this impact is attenuated if prepregnancy BMI is overweight rather than normal or if EGWG is extreme. These findings add to increasing evidence that effective treatment of prepregnancy obesity may help prevent adverse neonatal and obstetric outcomes. Although more studies are needed regarding the specific effects of prepregnancy weight and nutrition optimization, including the use of weight loss medications such as GLP-1 RAs, our data provides information to clinicians advising patients on obesity management prior to pregnancy.

## Figures and Tables

**Table 1:** Baseline characteristics.

	Normal (n = 64,156)	Overweight (n = 36,943)	Obesity (n = 28,516)
Age, y (mean (SD))	31.64 (5.35)	31.94 (5.28)	31.96 (5.31)
BMI (mean(SD))	22.02 (1.72)	27.14 (1.41)	34.90 (4.82)
Gestational Weight Gain (%)			
Excessive	20,763 (32.4)	21,821 (59.1)	16,223 (56.9)
Recommended	25,713 (40.1)	10,384 (28.1)	7364 (25.8)
Lower	17,680 (27.6)	4738 (12.8)	4929 (17.3)
Nulliparous (%)	29,162 (45.5)	14,634 (39.6)	10,846 (38.0)
Race/Ethnicity (%)			
Non-Hispanic White	32,115 (50.1)	13,864 (37.5)	9446 (33.1)
Non-Hispanic Black	4714 (7.3)	4963 (13.4)	6091 (21.4)
Hispanic	8948 (13.9)	8546 (23.1)	7439 (26.1)
Asian or Pacific Islander	10,471 (16.3)	4834 (13.1)	2246 (7.9)
Other	7908 (12.3)	4736 (12.8)	3294 (11.6)
Chronic Hypertension (%)	719 (1.1)	1122 (3.0)	1974 (6.9)
Public Insurance (%)	19,993 (31.2)	14,443 (39.1)	11,573 (40.6)

Data are presented as mean ±standard deviation, and n (%). For BMI, the mean (SD) are as follows: normal: 22.02 (1.72); overweight: 27.14 (1.41); and obese: 34.90 (4.82). All variables were statistically significant at *p* < 0.001 when compared between prepregnancy BMI categories.

**Table 2:** Adjusted relative risks of adverse maternal and neonatal outcomes comparing normal or overweight prepregnancy BMI to prepregnancy obesity with no change in gestational weight gain (GWG).

Outcome	Normal	Overweight
Maternal Outcomes		
Gestational Diabetes	0.37 (0.35, 0.38) *p* < 0.001	0.71 (0.69, 0.74) *p* < 0.001
Gestational Hypertension	0.54 (0.51, 0.57) *p* < 0.001	0.69 (0.65, 0.73) *p* < 0.001
Preeclampsia	0.45 (0.43, 0.48) *p* < 0.001	0.63 (0.61, 0.66) *p* < 0.001
Eclampsia	0.31 (0.19, 0.52) *p* < 0.001	0.48 (0.29, 0.78) *p* < 0.01
Operative Delivery	1.8 (1.65, 1.96) *p* < 0.001	1.43 (1.3, 1.56) *p* < 0.001
Cesarean Delivery	0.65 (0.63, 0.66) *p* < 0.001	0.78 (0.77, 0.8) *p* < 0.001
Shoulder Dystocia	0.7 (0.6, 0.8) *p* < 0.001	0.92 (0.8, 1.06)
Postpartum Hemorrhage	0.71 (0.65, 0.76) *p* < 0.001	0.88 (0.82, 0.96) *p* < 0.01
Neonatal Outcomes		
LGA Newborn	0.52 (0.49, 0.56) *p* < 0.001	0.69 (0.65, 0.73) *p* < 0.001
SGA Newborn	1.11 (1.05, 1.17) *p* < 0.001	1.12 (1.06, 1.19) *p* < 0.001
Stillbirth	0.45 (0.35, 0.59) *p* < 0.001	0.87 (0.67, 1.13)
NICU Admission	0.63 (0.6, 0.67) *p* < 0.001	0.81 (0.77, 0.86) *p* < 0.001
Mechanical Ventilation	0.43 (0.34, 0.54) *p* < 0.001	0.81 (0.65, 1)
RDS	0.37 (0.31, 0.43) *p* < 0.001	0.71 (0.61, 0.83) *p* < 0.001
Intraventricular Hemorrhage	0.35 (0.26, 0.48) *p* < 0.001	0.84 (0.63, 1.11)
Necrotizing Enterocolitis	0.23 (0.13, 0.39) *p* < 0.001	0.54 (0.31, 0.94) *p* < 0.05
HIE	0.67 (0.36, 1.27)	1.06 (0.56, 2)
Neonatal Sepsis	0.4 (0.29, 0.56) *p* < 0.001	0.71 (0.51, 0.98)
Umbilical Artery PH < 7	0.61 (0.51, 0.73) *p* < 0.001	0.84 (0.71, 1)

LGA = large for gestational age, SGA = small for gestational age, NICU = neonatal intensive care unit, RDS = respiratory distress syndrome, and HIE = hypoxic ischemic encephalopathy.

**Table 3:** Adjusted relative risks of adverse maternal and neonatal outcomes comparing normal (or overweight) prepregnancy BMI and excessive GWG to prepregnancy obesity and any GWG (or with recommended GWG).

Outcome	Normal + EGWG vs. Obese with Any GWG	Overweight + EGWG vs. Obese with Any GWG	Normal + EGWG vs. Obese with RGWG	Overweight + EGWG vs. Obese with RGWG
Maternal Outcomes				
Gestational Diabetes	0.3 (0.28, 0.32) *p* < 0.001	0.53 (0.51, 0.56) *p* < 0.001	0.25 (0.23, 0.27) *p* < 0.001	0.45 (0.42, 0.47) *p* < 0.001
Gestational Hypertension	0.64 (0.6, 0.69) *p* < 0.001	0.82 (0.77, 0.88) *p* < 0.001	0.7 (0.63, 0.77) *p* < 0.001	0.89 (0.81, 0.97) *p* < 0.05
Preeclampsia	0.54 (0.51, 0.57) *p* < 0.001	0.71 (0.67, 0.75) *p* < 0.001	0.59 (0.54, 0.64) *p* < 0.001	0.77 (0.71, 0.83) *p* < 0.001
Eclampsia	0.35 (0.18, 0.68) *p* < 0.01	0.33 (0.17, 0.65) *p* < 0.01	0.35 (0.15, 0.82) *p* < 0.05	0.34 (0.15, 0.78) *p* < 0.05
Operative Delivery	1.76 (1.6, 1.94) *p* < 0.001	1.45 (1.31, 1.61) *p* < 0.001	1.78 (1.52, 2.09) *p* < 0.001	1.47 (1.25, 1.73) *p* < 0.001
Cesarean Delivery	0.7 (0.69, 0.72) *p* < 0.001	0.83 (0.81, 0.85) *p* < 0.001	0.74 (0.71, 0.76) *p* < 0.001	0.87 (0.84, 0.9) *p* < 0.001
Shoulder Dystocia	0.87 (0.74, 1.03)	1.11 (0.96, 1.3)	0.87 (0.69, 1.11)	1.12 (0.89, 1.41)
Postpartum Hemorrhage	0.74 (0.67, 0.82) *p* < 0.001	0.84 (0.76, 0.92) *p* < 0.001	0.75 (0.65, 0.86) *p* < 0.001	0.85 (0.74, 0.97) *p* < 0.05
Neonatal Outcomes				
LGA Newborn	0.77 (0.72, 0.83) *p* < 0.001	0.96 (0.9, 1.03)	1.05 (0.93, 1.18)	1.3 (1.17, 1.46) *p* < 0.001
SGA Newborn	0.8 (0.74, 0.86) *p* < 0.001	0.83 (0.78, 0.9) *p* < 0.001	0.66 (0.6, 0.73) *p* < 0.001	0.69 (0.63, 0.76) *p* < 0.001
Stillbirth	0.26 (0.16, 0.43) *p* < 0.001	0.56 (0.39, 0.81) *p* < 0.01	0.18 (0.1, 0.32) *p* < 0.001	0.39 (0.25, 0.61) *p* < 0.001
NICU Admission	0.61 (0.58, 0.66) *p* < 0.001	0.76 (0.72, 0.81) *p* < 0.001	0.61 (0.55, 0.66) *p* < 0.001	0.75 (0.69, 0.82) *p* < 0.001
Mechanical Ventilation	0.34 (0.24, 0.49) *p* < 0.001	0.51 (0.38, 0.68) *p* < 0.001	0.35 (0.22, 0.53) *p* < 0.001	0.51 (0.34, 0.76) *p* < 0.01
RDS	0.23 (0.17, 0.31) *p* < 0.001	0.43 (0.34, 0.53) *p* < 0.001	0.18 (0.13, 0.24) *p* < 0.001	0.33 (0.25, 0.42) *p* < 0.001
Intraventricular Hemorrhage	0.28 (0.17, 0.46) *p* < 0.001	0.47 (0.31, 0.7) *p* < 0.001	0.21 (0.12, 0.38) *p* < 0.001	0.36 (0.22, 0.59) *p* < 0.001
Necrotizing Enterocolitis	0.04 (0.01, 0.33) *p* < 0.01	0.3 (0.13, 0.68) *p* < 0.01	0.04 (0, 0.28) *p* < 0.01	0.24 (0.09, 0.62) *p* < 0.01
HIE	0.57 (0.23, 1.37)	1 (0.49, 2.06)	0.62 (0.18, 2.12)	1.1 (0.36, 3.36)
Neonatal Sepsis	0.36 (0.22, 0.58) *p* < 0.001	0.46 (0.29, 0.71) *p* < 0.001	0.27 (0.15, 0.49) *p* < 0.001	0.35 (0.2, 0.6) *p* < 0.001
Umbilical Artery pH < 7	0.63 (0.5, 0.79) *p* < 0.001	1.04 (0.86, 1.26)	0.69 (0.5, 0.95) *p* < 0.05	1.14 (0.85, 1.53)

EGWG = excessive gestational weight gain, RGWG = recommended gestational weight gain, LGA = large for gestational age, SGA = small for gestational age, NICU = neonatal intensive care unit, RDS = respiratory distress syndrome, and HIE = hypoxic ischemic encephalopathy. *p* values adjusted for 19 comparisons using the Benjamini–Hochberg method.

**Table 4:** Adjusted relative risk of adverse maternal and neonatal outcomes comparing normal prepregnancy BMI and excessive GWG of 1–9 lbs, 10–19 lbs, and ≥20 lbs of GWG to obese prepregnancy BMI and recommended GWG.

Outcome	Normal + EGWG 1–9 lbs vs. Obese with RGWG	Normal + EGWG 10–19 lbs vs. Obese with RGWG	Normal + EGWG ≥20 lbs vs. Obese with RGWG	All Equal (*p*)
Maternal Outcomes				
Gestational Diabetes	0.25 (0.23, 0.27)	0.23 (0.21, 0.26)	0.3 (0.26, 0.35)	0.022
Gestational Hypertension	0.6 (0.54, 0.67)	0.72 (0.63, 0.83)	1.08 (0.93, 1.25)	<0.001
Preeclampsia	0.52 (0.47, 0.57)	0.58 (0.51, 0.65)	0.93 (0.82, 1.05)	<0.001
Eclampsia	0.33 (0.13, 0.85)	1.56 (0.5, 4.88)	0.04 (0.02, 0.08)	<0.001
Operative Delivery	1.87 (1.58, 2.2)	1.68 (1.39, 2.04)	1.61 (1.27, 2.04)	0.214
Cesarean Delivery	0.69 (0.66, 0.71)	0.78 (0.75, 0.82)	0.88 (0.83, 0.93)	<0.001
Shoulder Dystocia	0.8 (0.61, 1.04)	0.98 (0.72, 1.34)	0.99 (0.67, 1.46)	0.292
Postpartum Hemorrhage	0.72 (0.61, 0.84)	0.72 (0.6, 0.88)	0.93 (0.74, 1.16)	0.066
Neonatal Outcomes				
LGA Newborn	0.81 (0.71, 0.93)	1.2 (1.04, 1.39)	1.81 (1.55, 2.12)	<0.001
SGA Newborn	0.71 (0.64, 0.79)	0.62 (0.54, 0.71)	0.52 (0.43, 0.63)	0.003
Stillbirth	0.3 (0.15, 0.61)	0.27 (0.1, 0.72)	0.12 (0.03, 0.44)	0.434
NICU Admission	0.58 (0.52, 0.64)	0.64 (0.56, 0.73)	0.68 (0.57, 0.79)	0.079
Mechanical Ventilation	0.3 (0.18, 0.51)	0.39 (0.2, 0.74)	0.46 (0.21, 1.03)	0.57
RDS	0.19 (0.13, 0.27)	0.18 (0.11, 0.31)	0.12 (0.05, 0.3)	0.675
Intraventricular Hemorrhage	0.22 (0.11, 0.43)	0.13 (0.04, 0.43)	0.36 (0.13, 1.02)	0.418
Necrotizing Enterocolitis	0.05 (0.03, 0.09)	0.09 (0.03, 0.28)	0.05 (0.03, 0.08)	0.36
HIE	0.45 (0.13, 1.63)	0.34 (0.1, 1.24)	0.39 (0.07, 2.07)	0.917
Neonatal Sepsis	0.29 (0.14, 0.61)	0.56 (0.19, 1.68)	0.34 (0.06, 1.81)	0.573
Umbilical Artery pH < 7	0.7 (0.49, 1)	0.68 (0.43, 1.06)	0.68 (0.38, 1.23)	0.989

EGWG = excessive gestational weight gain, RGWG = recommended gestational weight gain, LGA = large for gestational age, SGA = small for gestational age, NICU = neonatal intensive care unit, RDS = respiratory distress syndrome, and HIE = hypoxic ischemic encephalopathy.

**Table 5:** Adjusted relative risk of adverse maternal and neonatal outcomes comparing overweight prepregnancy BMI and excessive GWG of 1–9 lbs, 10–19 lbs, and ≥20 lbs of GWG to obese prepregnancy BMI and recommended GWG.

Outcome	Overweight + EGWG 1–9 lbs vs. Obese with RGWG	Overweight + EGWG 10–19 lbs vs. Obese with RGWG	Overweight + EGWG ≥20 lbs vs. Obese with RGWG	All Equal (*p*)
Maternal Outcomes				
Gestational Diabetes	0.5 (0.46, 0.54)	0.43 (0.4, 0.47)	0.37 (0.33, 0.41)	<0.001
Gestational Hypertension	0.75 (0.67, 0.84)	0.91 (0.82, 1.03)	1.09 (0.97, 1.23)	<0.001
Preeclampsia	0.7 (0.64, 0.77)	0.72 (0.65, 0.79)	0.96 (0.87, 1.06)	<0.001
Eclampsia	0.56 (0.15, 2.09)	0.56 (0.19, 1.7)	0.61 (0.18, 2.04)	0.993
Operative Delivery	1.5 (1.25, 1.79)	1.59 (1.32, 1.91)	1.27 (1.03, 1.56)	0.062
Cesarean Delivery	0.81 (0.78, 0.84)	0.88 (0.84, 0.91)	0.96 (0.92, 1)	<0.001
Shoulder Dystocia	0.91 (0.69, 1.2)	1.08 (0.82, 1.44)	1.54 (1.16, 2.03)	<0.001
Postpartum Hemorrhage	0.88 (0.75, 1.03)	0.83 (0.7, 0.99)	0.83 (0.68, 1)	0.756
Neonatal Outcomes				
LGA Newborn	0.94 (0.82, 1.08)	1.3 (1.14, 1.49)	1.96 (1.72, 2.23)	<0.001
SGA Newborn	0.83 (0.74, 0.93)	0.64 (0.56, 0.73)	0.51 (0.44, 0.6)	<0.001
Stillbirth	0.47 (0.27, 0.81)	0.47 (0.26, 0.85)	0.25 (0.09, 0.66)	0.438
NICU Admission	0.69 (0.62, 0.77)	0.77 (0.69, 0.85)	0.84 (0.75, 0.95)	0.005
Mechanical Ventilation	0.36 (0.21, 0.62)	0.65 (0.4, 1.07)	0.58 (0.33, 1.02)	0.135
RDS	0.41 (0.3, 0.56)	0.28 (0.19, 0.42)	0.24 (0.15, 0.39)	0.079
Intraventricular Hemorrhage	0.31 (0.16, 0.61)	0.41 (0.21, 0.8)	0.37 (0.17, 0.81)	0.796
Necrotizing Enterocolitis	0.23 (0.08, 0.68)	0.63 (0.14, 2.83)	0.53 (0.1, 2.82)	0.41
HIE	1.37 (0.44, 4.23)	0.9 (0.26, 3.12)	0.59 (0.14, 2.46)	0.446
Neonatal Sepsis	0.46 (0.2, 1.04)	0.45 (0.18, 1.13)	0.33 (0.14, 0.78)	0.811
Umbilical Artery pH < 7	1.12 (0.8, 1.57)	1.06 (0.74, 1.52)	1.27 (0.88, 1.85)	0.617

EGWG = excessive gestational weight gain, RGWG = recommended gestational weight gain, LGA = large for gestational age, SGA = small for gestational age, NICU = neonatal intensive care unit, RDS = respiratory distress syndrome, and HIE = hypoxic ischemic encephalopathy.

**Table 6:** Adjusted relative risks of adverse maternal and neonatal outcomes in term pregnancies comparing normal or overweight prepregnancy BMI with prepregnancy obesity with any gestational weight gain.

Outcome	Normal + EGWG vs. Obese with Any GWG	Overweight + EGWG vs. Obese with Any GWG
Maternal Outcomes		
Gestational Diabetes	0.3 (0.28, 0.32) *p* < 0.001	0.54 (0.51, 0.57) *p* < 0.001
Gestational Hypertension	0.63 (0.59, 0.68) *p* < 0.001	0.81 (0.76, 0.87) *p* < 0.001
Preeclampsia	0.59 (0.56, 0.63) *p* < 0.001	0.75 (0.71, 0.8) *p* < 0.001
Eclampsia	0.45 (0.22, 0.93) *p* < 0.05	0.39 (0.18, 0.84) *p* < 0.05
Operative Delivery	1.7 (1.53, 1.88) *p* < 0.001	1.42 (1.28, 1.58) *p* < 0.001
Cesarean Delivery	0.71 (0.69, 0.73) *p* < 0.001	0.83 (0.81, 0.85) *p* < 0.001
Shoulder Dystocia	0.82 (0.69, 0.97) *p* < 0.05	1.06 (0.91, 1.24)
Postpartum Hemorrhage	0.79 (0.71, 0.88) *p* < 0.001	0.93 (0.84, 1.03)
Neonatal Outcomes		
LGA Newborn	0.77 (0.71, 0.83) *p* < 0.001	0.96 (0.9, 1.03)
SGA Newborn	0.82 (0.75, 0.88) *p* < 0.001	0.84 (0.78, 0.91) *p* < 0.001
Stillbirth	0.81 (0.37, 1.75)	1.14 (0.57, 2.27)
NICU Admission	0.77 (0.71, 0.84) *p* < 0.001	0.91 (0.84, 0.98) *p* < 0.05
Mechanical Ventilation	0.55 (0.32, 0.94) *p* < 0.05	1.18 (0.77, 1.8)
RDS	1.4 (0.53, 3.7)	0.84 (0.27, 2.6)
Intraventricular Hemorrhage	1.41 (0.39, 5.06)	2.5 (0.8, 7.85)
Necrotizing Enterocolitis	---	---
HIE	0.54 (0.17, 1.66)	1.19 (0.5, 2.83)
Neonatal Sepsis	0.83 (0.39, 1.78)	1.01 (0.49, 2.07)
Umbilical Artery pH < 7	0.66 (0.52, 0.84) *p* < 0.01	1.13 (0.92, 1.37)

EGWG = excessive gestational weight gain, LGA = large for gestational age, SGA = small for gestational age, NICU = neonatal intensive care unit, RDS = respiratory distress syndrome, and HIE = hypoxic ischemic encephalopathy. *p* values adjusted for 18 comparisons using the Benjamini–Hochberg method. Adjusted relative risks were not calculated for necrotizing enterocolitis due to the low sample size resulting in an unstable logistic regression model.

## Data Availability

The original data presented in this study is available upon reasonable request.

## Supplementary Materials

The following supporting information can be downloaded at: https://www.mdpi.com/article/10.3390/jcm15176858/s1, There are six Supplementary tables and 2 figures included; Table S1: Comparison of Covariates and Outcomes in data based on Missingness of BMI and GWG; Table S2: Balance of covariates in pre-pregnancy BMI categories for pregnancies with recommended gestational weight gain; Table S3: Balance of covariates in pre-pregnancy BMI categories for pregnancies with excessive gestational weight gain; Table S4: Balance of covariates in pre-pregnancy BMI categories for pregnancies with lower than recommended gestational weight gain; Table S5: Balance of covariates for pregnancies based on combinations of pre-pregnancy BMI and Gestational Weight Gain; Table S6: Balance of covariates for pregnancies based on combinations of pre-pregnancy BMI and gestational weight gain including subgroups of excessive gestational weight gain; Figure S1: Average Predicted Risk and 95% Confidence Interval of Adverse Maternal Outcomes Among Obese Women Based on Propensity Score Weighted Logistic Regression; Figure S2: Average Predicted Risk and 95% Confidence Interval of Adverse Neonatal Outcomes Among Obese Women Based on Propensity Score Weighted Logistic Regression.
