# Supplementary material for: Prepregnancy Obesity Versus Excessive Gestational Weight Gain: A Comparative Evaluation of Adverse Outcomes
Source: J Clin Med. 2026 Sep 4;15(17):6858. doi: 10.3390/jcm15176858 (PMC13566158; doi:10.3390/jcm15176858)
Supplement: Supplementary file 1 [file jcm-15-06858-s001.zip › overall_analysis_SS Edits 8-4-26 DK 81626.pdf]

Table S1: Comparison of Covariates and Outcomes in data based on Missingness of BMI and GWG

	**Not Missing** (n = 129,929)	**Missing** (n = 20,094)	**Difference**
**Missing (mean (SD))**	31.80 (5.33)	31.77 (5.29)	0.00
**Maternal Age (%)**	75069 (57.9)	11943 (59.7)	-0.02
**Nulliparous (%)**	3821 (2.9)	439 (2.2)	0.01
**Race/Ethnicity (%)**			
Non-Hispanic White	55565 (42.8)	9012 (44.8)	-0.02
Non-Hispanic Black	15797 (12.2)	1887 (9.4)	0.03
Hispanic	24995 (19.2)	3833 (19.1)	0.00
Asian or Pacific Islander	17587 (13.5)	2503 (12.5)	0.01
Other	15985 (12.3)	2859 (14.2)	-0.02
**Chronic Hypertension (%)**	46049 (35.5)	6975 (34.8)	0.01
**Public Insurance (%)**	15119 (11.6)	2092 (10.4)	0.01
**Gestational Diabetes (%)**	7282 (5.6)	759 (3.8)	0.02
**Gestational Hypertension (%)**	10294 (7.9)	1458 (7.3)	0.01
**Preeclampsia (%)**	97 (0.1)	23 (0.1)	0.00
**Eclampsia (%)**	4875 (3.8)	687 (3.4)	0.00
**Operative Delivery (%)**	43699 (33.6)	6011 (29.9)	0.04
**Cesarean Delivery (%)**	1368 (1.1)	77 (0.4)	0.01
**Shoulder Dystocia (%)**	4052 (3.1)	499 (2.5)	0.01
**Postpartum Hemorrhage (%)**	5770 (4.4)	828 (4.1)	0.00
**LGA newborn (%)**	10037 (7.7)	1581 (7.9)	0.00
**SGA newborn (%)**	376 (0.3)	73 (0.4)	0.00
**Stillbirth (%)**	9183 (7.1)	1178 (5.9)	0.01
**NICU Admission (%)**	509 (0.4)	75 (0.4)	0.00
**Mechanical Ventilation (%)**	1022 (0.8)	123 (0.6)	0.00
**RDS (%)**	284 (0.2)	32 (0.2)	0.00
**Intraventricular Hemorrhage (%)**	79 (0.1)	8 (0.0)	0.00
**Necrotizing Enterocolitis (%)**	67 (0.1)	10 (0.0)	0.00
**HIE (%)**	232 (0.2)	27 (0.1)	0.00
**Neonatal Sepsis (%)**	813 (0.9)	116 (0.9)	0.00
**Umbilical artery pH <7 (%)**	9252 (7.1)	1357 (6.8)	0.00
LGA = Large for gestational age, SGA = Small for gestational age, NICU = Neonatal intensive care unit, RDS = Respiratory Distress Syndrome, HIE = Hypoxic Ischemic Encephalopathy			

Difference for Maternal Age is the standardized mean Difference.

Table S2: Balance of covariates in pre-pregnancy BMI categories for pregnancies with recommended gestational weight gain

**Variable**	**Type**	**Maximum Difference Unadjusted**	**Maximum Difference Adjusted**
Nulliparous	Binary	0.110	0.003
Non-Hispanic White	Binary	0.214	0.005
Non-Hispanic Black	Binary	0.146	0.011
Hispanic	Binary	0.150	0.003
Asian or Pacific Islander	Binary	0.077	0.001
Other Race/Ethnicity	Binary	0.018	0.003
CHTN	Binary	0.064	0.011
Public Insurance	Binary	0.146	0.020
Maternal Age *P<0.05*	Continuous	0.056	0.035
CHTN = Chronic Hypertension			

P<0.05 Standardized Difference. Differences in binary variables are not standardized.
Effective sample sizes:
Unadjusted: Obese: 7,364 Overweight: 10,384 Normal: 25,713
Adjusted: Obese: 7,364 Overweight: 8,869.21 Normal: 9,029.2

Table S3: Balance of covariates in pre-pregnancy BMI categories for pregnancies with excessive gestational weight gain

**Variable**	**Type**	**Maximum Difference Unadjusted**	**Maximum Difference Adjusted**
Nulliparous	Binary	0.103	0.002
Non-Hispanic White	Binary	0.162	0.001
Non-Hispanic Black	Binary	0.117	0.002
Hispanic	Binary	0.106	0.001
Asian or Pacific Islander	Binary	0.057	0.001
Other Race/Ethnicity	Binary	0.007	0.001
CHTN	Binary	0.052	0.006
Public Insurance	Binary	0.072	0.014
Maternal Age*P<0.05*	Continuous	0.092	0.016
CHTN = Chronic Hypertension			

P<0.05 Standardized Difference. Differences in binary variables are not standardized.
Effective sample sizes:
Unadjusted: Obese: 16,223 Overweight: 21,821 Normal: 20,763
Adjusted: Obese: 16,223 Overweight: 19,649.53 Normal: 11,880.46

Table S4: Balance of covariates in pre-pregnancy BMI categories for pregnancies with lower than recommended gestational weight gain

**Variable**	**Type**	**Maximum Difference Unadjusted**	**Maximum Difference Adjusted**
Nulliparous	Binary	0.073	0.005
Non-Hispanic White	Binary	0.184	0.007
Non-Hispanic Black	Binary	0.164	0.007
Hispanic	Binary	0.127	0.002
Asian or Pacific Islander	Binary	0.122	0.002
Other Race/Ethnicity	Binary	0.028	0.003
CHTN	Binary	0.068	0.013
Public Insurance	Binary	0.142	0.014
Maternal Age*P<0.05*	Continuous	0.123	0.012
CHTN=chronic hypertension.			

P<0.05 Standardized Difference. Differences in binary variables are not standardized.
Effective sample sizes:
Unadjusted: Obese: 4,929 Overweight: 4,738 Normal: 17,680
Adjusted: Obese: 4,929 Overweight: 4,037.94 Normal: 6,872.07

Table S5: Balance of covariates for pregnancies based on combinations of pre-pregnancy BMI and Gestational Weight Gain

**Variable**	**Type**	**Maximum Difference Unadjusted**	**Maximum Difference Adjusted**
Nulliparous	Binary	0.191	0.013
Non-Hispanic White	Binary	0.281	0.009
Non-Hispanic Black	Binary	0.176	0.008
Hispanic	Binary	0.159	0.005
Asian or Pacific Islander	Binary	0.131	0.003
Other Race/Ethnicity	Binary	0.035	0.005
CHTN	Binary	0.070	0.013
Public Insurance	Binary	0.228	0.019
Maternal Age *P<0.05*	Continuous	0.165	0.048
CHTN=chronic hypertension.			

P<0.05 Standardized Difference. Differences in binary variables are not standardized.
Effective sample sizes:
Unadjusted: Recommended GWG + Obese: 7,364 Normal: 25,713 Overweight: 10,384
Adjusted: Recommended GWG + Obese: 7,364 Normal: 9,353.35 Overweight: 8,888.75
Unadjusted: Excessive GWG + Obese: 16,223 Normal: 20,763 Overweight: 21,821
Adjusted: Excessive GWG + Obese: 15,581.07 Normal: 9,053.02 Overweight: 18,029.14
Unadjusted: Lower than Recommended GWG + Obese: 4,929 Normal: 17,680 Overweight: 4,738
Adjusted: Lower than Recommended GWG + Obese: 4,864.935 Normal: 8,089.68 Overweight: 4,086.07

Table S6: Balance of covariates for pregnancies based on combinations of pre-pregnancy BMI and gestational weight gain including subgroups of excessive gestational weight gain

**Variable**	**Type**	**Maximum Difference Unadjusted**	**Maximum Difference Adjusted**
Nulliparous	Binary	0.253	0.027
Non-Hispanic White	Binary	0.281	0.016
Non-Hispanic Black	Binary	0.176	0.012
Hispanic	Binary	0.159	0.016
Asian or Pacific Islander	Binary	0.142	0.008
Other Race/Ethnicity	Binary	0.035	0.009
CHTN	Binary	0.070	0.021
Public Insurance	Binary	0.228	0.021
Maternal Age*P<0.05*	Continuous	0.300	0.074
CHTN=chronic hypertension.			

P<0.05 Standardized Difference. Differences in binary variables are not standardized.
Effective sample sizes:
Unadjusted: Recommended GWG + Obese: 7,364 Normal: 25,713 Overweight: 10,384
Adjusted: Recommended GWG + Obese: 7,364 Normal: 9,354.96 Overweight: 8,888.85
Unadjusted: Excessive GWG 1-9 lbs + Obese: 6,190 Normal: 12,488 Overweight: 9,403
Adjusted: Excessive GWG 1-9 lbs + Obese: 6,128.944 Normal: 4,791.77 Overweight: 7,968.29
Unadjusted: Excessive GWG 10-19 lbs + Obese: 5,256 Normal: 5,552 Overweight: 7,158
Adjusted: Excessive GWG 10-19 lbs + Obese: 5,016.998 Normal: 2,735.89 Overweight: 5,717.88
Unadjusted: Excessive GWG >=20 lbs + Obese: 4,777 Normal: 2,723 Overweight: 5,260
Adjusted: Excessive GWG >=20 lbs + Obese: 4,260.096 Normal: 1,293.41 Overweight: 4,013.98
Unadjusted: Lower than Recommended GWG + Obese: 4,929 Normal: 17,680 Overweight: 4,738
Adjusted: Lower than Recommended GWG + Obese: 4,864.855 Normal: 8,092.66 Overweight: 4,086.35
